# Factors associated with first suicide attempt vs. re-attempt in children and adolescents: A systematic review and meta-analysis

**DOI:** 10.1192/j.eurpsy.2023.1220

**Published:** 2023-07-19

**Authors:** S. Abascal-Peiró, A. Alacreu-Crespo, I. Peñuelas-Calvo, B. Ezquerra-de la Cruz, L. Jiménez-Muñoz, E. Baca-García, A. Porras-Segovia

**Affiliations:** 1 Psychiatry, Hospital Universitario Rey Juan Carlos, Madrid; 2 Psychology and Sociology, Area of Personality, Assessment and Psychological Treatment, University of Zaragoza, Teruel; 3 Psychiatry, Hospital Universitario 12 de Octubre; 4 Psychiatry, Jimenez Diaz Foundation Health Research Institute (IIS); 5 Psychiatry, Autonomous University of Madrid, Madrid, Spain; 6 Psychiatry, Imperial College, London, United Kingdom

## Abstract

**Introduction:**

Suicide among children, adolescents and young adults is a major health problem, as it represents the fourth leading cause of death among people aged 15-29 (WHO, 2022). A recent study showed that the years of potential life lost (YPLL) due to suicide in 2018 were 1,344,552, which is very close to the 1,591,487 YPLL caused by COVID-19 in the year 2020 (Porras-Segovia et al, 2021). In the recent years, there is a growing interest in suicide prevention research in differentiating attempter profiles in terms of lifetime suicide attempts. Specifically, studies suggest that there may be meaningful differences concerning risk factors between patients with a history of one versus multiple suicide attempts. Multiple attempters (MA) show more suicidal ideation, depressive symptoms and hopelessness than single attempters (SA) (Esposito et al, 2003; Goldston et al, 1998).

**Objectives:**

We aimed to answer the question ‘What are the factors associated with attempting suicide for the first time and are they different from the factors associated with re-attempting suicide in children and adolescents?’

**Methods:**

We conducted a systematic literature search in four databases. Article selection and data extraction according to a predefined protocol, including bias risk assessment, were performed by independent peer reviewers. Due to the different way to present data in the studies effect sizes were pre-calculated to standard mean differences (SMD). Random effects model was used to calculate the pooled effect size for all meta-analysis. Publication bias was assessed using funnel plots.

**Results:**

14 studies were included in the systematic review, and 13 in the meta-analysis. Original articles used in the meta-analysis included a total of 4286 participants of whom 1579 were multiple suicide-attempters and 2707 single suicide attempters.

MAs showed significantly higher proportion of anxiety disorders (SMD = 0.387, 95%CI [0.09, 0.68], p < 0.022), alcohol abuse disorder (SMD = 0.382, 95%CI [0.07, 0.70], p < 0.036) and substance abuse disorder (SMD = 0.526, 95%CI [0.21, 0.84], p <0.013) than SAs. Mean depression severity was higher among MAs than SAs (SMD = 0.515, 95%CI [0.17, 0.86], p < 0.011). MAs showed higher impulsivity (SMD = 0.28, 95%CI [-0.03, 0.60], p < 0.068) and aggressiveness (SMD = 0.688, 95%CI [0.42, 0.96], p < 0.00) than SAs. Hopelessness (SMD = 0.482, 95%CI [0.06, 0.91], p < 0.03) and suicidal ideation (SMD = 0.399, 95%CI [0.34, 0.46], p < 0.007) was significantly higher in MA.

**Conclusions:**

On the basis of the current results, multiple attempters may represent a distinct patient population in terms of being a more severe clinical profile. This can provide the basis of stronger suicide prevention and vigilance programs focused in this suicidal phenotype.

**Disclosure of Interest:**

None Declared

